# Odor–Taste–Texture Interactions as a Promising Strategy to Tackle Adolescent Overweight

**DOI:** 10.3390/nu13103653

**Published:** 2021-10-19

**Authors:** Cristina Proserpio, Elvira Verduci, Gianvincenzo Zuccotti, Ella Pagliarini

**Affiliations:** 1 Sensory & Consumer Science Lab (SCS_Lab), Department of Food, Environmental and Nutritional Sciences (DeFENS), University of Milan, 20133 Milan, Italy; cristina.proserpio@unimi.it; 2 Department of Pediatrics, Vittore Buzzi Children’s Hospital, University of Milan, 20154 Milan, Italy; Elvira.verduci@unimi.it (E.V.); gianvincenzo.zuccotti@unimi.it (G.Z.); 3 Department of Health Sciences, University of Milan, 20146 Milan, Italy; 4 Department of Biomedical and Clinical Science “L. Sacco”, University of Milan, 20157 Milan, Italy

**Keywords:** sensory perception, cross-modal interactions, food liking, dessert, aromas, thickener agent

## Abstract

The adolescence period is characterized by a considerable risk to weight gain due to the high consumption of food rich in sugar. A promising strategy to reduce sugar consumption may lie in exploiting the ability of our senses to interact to each other (cross-modal interactions). The aims were to investigate the cross-modal interactions and gustatory function in normal-weight and overweight adolescents. Fifty adolescents (25 overweight and 25 normal-weight) were involved. Subjects rated liking and attribute intensity in pudding samples obtained by adding vanilla aroma (0.1%; 0.3%), butter aroma (0.05%; 0.1%) or a thickener agent (1%; 1.5%) to a base formulation. The gustatory function was also measured through the “taste strips” methodology. Overweight adolescents were found to have a significantly (*p* < 0.001) worse ability to correctly identify all tastes. Cross-modal interactions occurred differently according to their body mass index, with a significant increase (*p* < 0.05) in sensory desirable characteristics (e.g., sweet and creaminess) due to aroma addition, especially in overweight subjects. Furthermore, butter aroma significantly increased hedonic responses only in overweight subjects. Tricking our senses in the way of perceiving sensory attributes could be a promising strategy to develop innovative food formulations with a reduced sugar amount, which will lead to a potential decrease in caloric intake and help to tackle the obesity epidemic.

## 1. Introduction

Adolescence, according to WHO, is the period from 10 to 19 years old, and is recognized as a susceptibility phase in terms of dietary behavior and decision making [1]. During this specific time in life, some interplaying factors could be associated with food consumption above physiological needs, leading to an increase in weight. Indeed, in this time in life, the regulatory processes at the basis of eating behavior, which help in restraining highly palatable and calorie-dense food ingestion, are underdeveloped due to the prefrontal cortex development. This specific area is then linked to the self-regulation process [2]. Moreover, it is widely reported that adolescents are motivated by food rewards and emotional eating [3], and respond to a positive and negative emotional status by usually consuming sweet foods [4].

The high consumption of sweet foods is directly related to the phenomenon of weight gain in childhood and adolescence, and could persist in adulthood [5]. Besides contributing to the increasing prevalence of obesity, excessive sugar intake is also associated with other diseases, such as type 2 diabetes [6]. It is also worth noting that the actual prevalence of overweight and obesity in young subjects represents both a health issue and a sustainability issue, since the amount of food eaten that is above the physiological needs have to be considered as food waste [7]. Even if it is clear that there is a necessity to counteract the obesity epidemic, the traditional approaches applied (i.e., dietary approaches) seem to not be as efficient as expected. Therefore, new strategies have to be identified to promote a sugar intake reduction. One strategy that could be applied is in the development of new food products with a reduced sugar amount that are still satisfying from a sensory point of view for the consumers.

In this context, the use of non-nutritive sweeteners, both natural and synthetic, has been the most common strategy to reduce the sugar content of food [8,9]. However, the use of these sweeteners could lead to unpleasant sensory characteristics, such as a bitter taste and metallic off-flavor [8,10]. A promising strategy to reduce the sugar concentration could lie in the use of cross-modal interactions, namely the ability of our senses to interact with each other, in new food formulations. In this context, literature data revealed that the addition of aromas signaling sweet-tasting products, such as caramel or fruit notes, increased the sweetness perception in model solutions, e.g., [11,12]. This odor–taste interaction has also been highlighted in model foods, e.g., [13,14]. Recently, [15] showed that a vanilla odor was able to enhance the perceived sweetness and the subsequent liking in a vanilla milk dessert with a lower concentration of sugar in a group of children. 

Besides the overconsumption of sweet food, another aspect that is directly related to adolescents’ weight gain is the amount of food ingested. A sensory interaction that could be worthy of interest in trying to affect the amount eaten involves the texture dimension. Indeed, thicker foods and beverages may create a higher expectation of satiety and may consequently decrease food intake [16]. Literature data showed that texture modulation from thinner to thicker was able to reduce the eating rate, as well as the food intake, in adults [17,18,19], as well as in children [20]. However, it should be considered that changes in texture perception could lead to a limited access of both volatile and tastant compounds [21,22,23]. Nevertheless, it was also demonstrated that odors with fatty notes, such as coconut and butter aromas, were able to increase the perceived thickness in low-fat stirred yoghurts [24].

Even if several studies have been conducted on sensory interactions, few of them have taken into account the different ability to perceived sensory qualities according to the body mass index (BMI) of the subjects involved. Overweight and obese subjects, although literature data are very controversial, have been reported to have a distorted taste e.g., [22,25] and odor perception e.g., [26,27] compared with normal-weight subjects. These differences in the ability of perceiving taste and odor qualities could also be translated in how the sensory interactions occur in subjects with different nutritional status. Accordingly, it was demonstrated that the addition of vanilla and butter aromas, generally associated with a high carbohydrates/fat content and pleasant food, increased the perception of other desirable sensory attributes, such as a sweet taste and overall hedonic response in a group of massively obese adults [28,29]. To our knowledge, no evidence is reported in adolescents, even if they represent an interesting target of consumers, as the overweight and obesity status could persist until adulthood.

From this perspective, the aim of the present study was to go further in the study of cross-modal sensory interactions in normal-weight and overweight adolescents. In particular, the effects of aromas and the addition of a thickener agent to a sweet model food (vanilla pudding) on sensory attributes and hedonic perception were investigated. The subjects involved were also characterized for their gustatory function in order to assess differences in taste perception according to BMI. 

## 2. Materials and Methods

### 2.1. Subjects

A total of fifty adolescents were involved. Twenty-five subjects were overweight (BMI = 28 ± 0.2 kg/m^2^) and twenty-five were normal-weight (BMI = 20 ± 0.3 kg/m^2^), according to WHO reference values. Overweight subjects were recruited among patients referred to the Department of Pediatrics, San Paolo Hospital (Milan, Italy). The population sample size is rather small due to the necessity of interrupting the sensory data collection as a consequence of the COVID-19 pandemic. However, the sample size is comparable to other studies with similar purposes, e.g., [30]. The exclusion criteria were as follows: subjects with food allergies, subjects who experienced ageusia or subjects who were on a medical treatment that could modify taste and odor perception. Only adolescents aged between 10 to 19 years old were involved. Every subject was asked for informed consent before making the assessments. All of the evaluations were performed in a quiet room and all of the participants were tested at the same time (10:30–12:30). They were asked to refrain from consuming anything but water for 2 h before the test (hungry state). The present study was performed according to the principles established by the Declaration of Helsinki after the protocol was approved by the Institutional Ethics Committee (amendment to ethic approval n° 210, dated: 28 February 2018).

### 2.2. Samples Preparation

A commercial pudding powder (ingredients: sugar, modified starch, dextrose, carrageenan, flavorings and coloring; Elah Dofour S.p.a., Novi Ligure, Italy) was used to prepare a control sample (Base_p) by adding 75 g of powder to 350 mL of skimmed milk. The milk and the powder were put into a tall, narrow container and mixed vigorously and without interruption with an immersion blender for 15 s. Experimental samples were obtained by adding vanilla aroma (0.1%; 0.3%; Flavourart, Oleggio, Italy) and by adding either butter aroma (0.05%; 0.1%; Flavourart, Oleggio, Italy) to the milk or by adding a thickener agent, specifically xanthan gum, to the pudding powder (1%, 1.5%; Sigma Aldrich, S.p.a., Milano, Italy). Aromas and gum concentrations, based on previous studies [28,29], were chosen through pilot triangle tests to elicit subtle but detectable differences between the Base_p and the added samples. Pudding samples were prepared on the day of the experimental sessions and were presented in triad (first triad: Base_p, A_Vanilla1 and A_Vanilla2; second triad: Base_p, A_Butter1 and A_Butter2 and third triad: Base_p, T_Xanthan1 and T_Xanthan2). 

### 2.3. Gustatory Function Assessment

“Taste strips” method [31], which shows a good test–retest reliability [32], was used to investigate the gustatory function of the adolescents involved. A total number of 18 paper strips (Taste Strips, Burghart, Wedel, Germany) were used. Filter papers have a length of 8 cm and a tip area of 2 cm^2^, and are impregnated with 4 concentrations of each of the 4 basic tastes (sweet: 0.4, 0.2, 0.1, 0.05 g/mL sucrose; sour: 0.3, 0.165, 0.09, 0.05 g/mL citric acid; salty: 0.25, 0.1, 0.04, 0.016 g/mL sodium chloride; bitter: 0.006, 0.0024, 0.0009, 0.0004 g/mL quinine hydrochloride). Two blank strips are not impregnated with a compound. The taste strips were presented in increasing concentrations with taste stimuli randomized across participants. Each subject placed the strip on the tongue and was asked to identify the perceived taste quality by choosing among five alternative responses (sweet, sour, salty, bitter, no taste). Prior to tasting, taste qualities were explained to the adolescents and they were instructed to rinse their mouth with water before assessment of each strip.

### 2.4. Overall Liking and Sensory Attributes Ratings Assessment 

Participants were asked to taste the products monadically and to express their liking scores using a 10 cm visual analogue scale (VAS) anchored by the extremes “extremely disliked” (rated 0) and “extremely liked” (rated 10). After a short break, they had to rate how they perceived the intensity of selected attributes (sweet, vanilla and butter flavor, creaminess) for each sample using a VAS anchored by the extremes “not at all” (rated 0) and “very much” (rated 10).

### 2.5. Experimental Design

Subjects attended two separate sessions. In both sessions, subjects were seated separately in a quiet room under similar light conditions. During the first session, the adolescents involved performed the gustatory function evaluation through the “taste strips” method. Between the strips, subjects were instructed to rinse their palate with water and to wait approximately 1 min, or more if needed, in order to have no residual taste in their mouth. This session took approximately 30 min. During the second session, participants had to evaluate liking and attribute intensity ratings of pudding samples. The pudding samples within each triad were randomly presented, whereas the presentation order of the triad was fixed for all subjects. Samples modified with vanilla aroma were always provided first, followed by samples added with butter aroma and then the samples modified in texture with the xanthan gum. This choice was due to the highly saturating nature of the texture-modified samples. Samples were coded with different three-digit numbers in each of the tests. All stimuli were prepared on the same day of the session and were presented at room temperature (20–22 °C), and 20 g of each sample was provided to the subjects. This session took approximately 45 min.

### 2.6. Data Analysis

A total taste score (TTS) was calculated as a sum of all correctly identified taste strips of the qualities sweet, sour, salty and bitter, yielding a range of 0–16. ANOVA model was performed considering BMI (normal-weight, overweight) as independent variables and the TTS as dependent variable.

ANOVAs were performed considering BMI (normal-weight, overweight), samples (Base_p, A_Vanilla1 and A_Vanilla2; Base_p, A_Butter1, A_Butter2; Base_p, T_Xanthan1, T_Xanthan2) and their interactions as independent variables, while sensory attribute ratings (sweetness, vanilla and butter flavor, creaminess) and liking ratings were considered as dependent variables. When a significant difference (*p* < 0.05) was found, least significant difference (LSD) post hoc test was used. These statistical analyses were performed using IBM SPSS Statistics for Windows, Version 26.0 (IBM Corp., Armonk, NY, USA).

## 3. Results

### 3.1. Gustatory Function

The ANOVA results revealed a significant BMI effect on the gustatory function. As reported in Table 1, overweight adolescents were found to have the worst performance in correctly identify all of the taste qualities compared with normal-weight adolescents. 

Generally, the normal-weight adolescents also performed better in identifying the lowest concentrations compared to overweight subjects. The taste stimulus that was more difficult to be identified at the lowest concentration in both groups of subjects was the sour taste stimulus. 

### 3.2. Sensory Attributes Ratings

#### 3.2.1. Samples Modified with Vanilla Aroma

**Figure 1:**
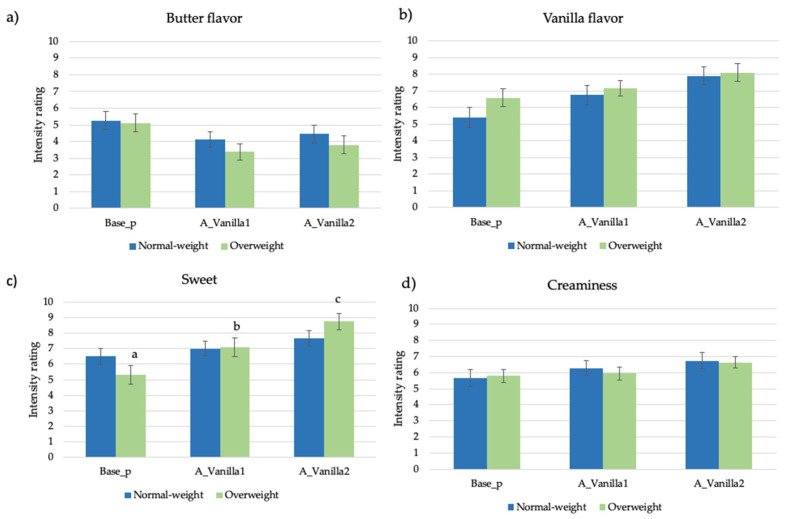
(**a**–**d**). Mean attribute intensity ratings (±SEM) by samples and BMI in pudding samples added with vanilla aroma. Different letters in the same group of subjects show significant differences (*p* < 0.05) according to post hoc test.

The attribute intensity ratings provided to the pudding samples modified with the vanilla aroma by normal-weight and overweight adolescents are reported in Figure 1a–d.

A significant interaction effect (sample × BMI; F = 3.05, *p* = 0.05) was found only in the sweet taste perception (Figure 1c). In particular, even if the addition of vanilla generally leads to an increase in the sweet taste as well as in the vanilla flavor perception in both groups of subjects, the samples added with the aroma were only perceived as being significantly sweeter (A_Vanilla1 = 7.1 ± 0.3; A_Vanilla2 = 8.8 ± 0.3) compared to Base_p (5.3 ± 0.4) in the overweight group of adolescents.

#### 3.2.2. Samples Modified with Butter Aroma

**Figure 2:**
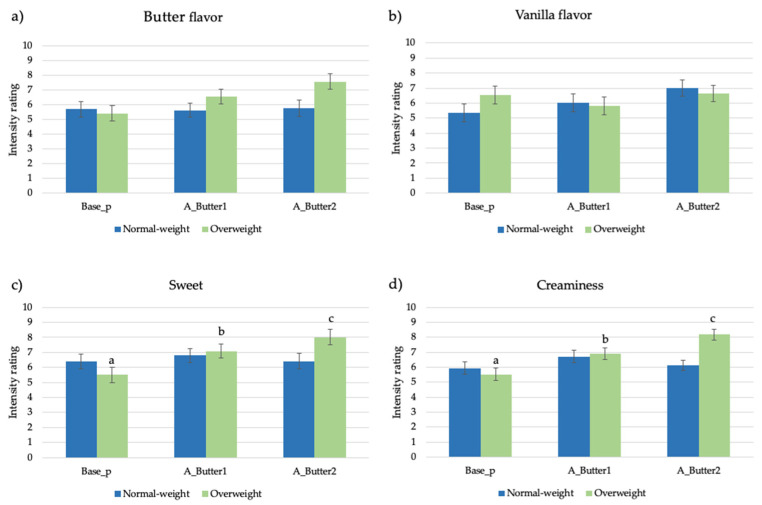
(**a**–**d**). Mean attribute intensity ratings (±SEM) by samples and BMI in pudding samples added with butter aroma. Different letters in the same group of subjects show significant differences (*p* < 0.05) according to post hoc test.

The attribute intensity ratings provided to the pudding samples modified with the butter aroma by normal-weight and overweight adolescents are reported in Figure 2a–d.

A significant interaction effect (sample × BMI) was found in the sweet taste (F = 3.3, *p* = 0.04) and creaminess perception (F = 5.8, *p* = 0.004) (Figure 2c,d). In particular, overweight adolescents perceived samples A_Butter1 and A_Butter2 as being significantly sweeter (7.1 ± 0.5, 8.0 ± 0.4, respectively) and creamier (6.9 ± 0.3, 8.2 ± 0.4) compared to the unmodified sample (sweetness: 5.5 ± 0.5, creaminess: 5.5 ± 0.4), whereas no differences were found in the normal-weight group. Moreover, overweight adolescents provided generally significant (F = 3.92, *p* = 0.05) higher scores (6.5 ± 0.2) for butter flavor perception compared with the control group (5.7 ± 0.3). 

#### 3.2.3. Samples Modified with Thickener Agent

**Figure 3:**
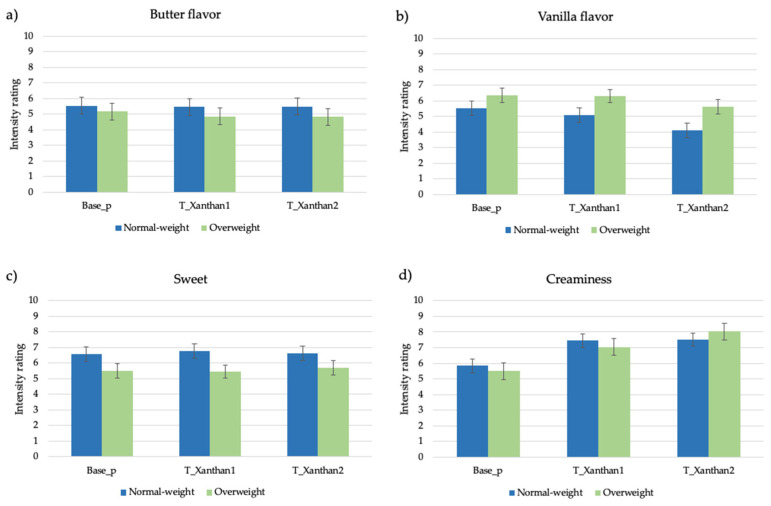
(**a**–**d**). Mean attribute intensity ratings (±SEM) by samples and BMI in pudding samples added with thickener agent.

The attribute intensity ratings provided to the pudding samples modified with the thickener agent by normal-weight and overweight adolescents are reported in Figure 3a–d. No BMI × sample effect was found in attribute intensity ratings. As expected, samples T_Xanthan1 and T_Xanthan2 were generally perceived as being significantly creamier (F = 14.4, *p* < 0.0001; 7.2 ± 0.3; 7.8 ± 0.3, respectively) than the unmodified pudding (5.7 ± 0.3) in both groups of subjects. Moreover, the xanthan gum addition leads to a significant decrease in vanilla flavor (F = 3.39, *p* = 0.04), with the sample added with the highest thickener agent amount perceived as being less flavored (4.8 ± 0.3) compared with T_Xanthan1 (5.7 ± 0.3) and Base_p (5.9 ± 0.3), which did not significantly differ from each other. 

### 3.3. Overall Liking

The hedonic evaluation for the samples modified by the vanilla aroma addition highlighted a significant sample effect (F = 10.23, *p* < 0.0001), with significant higher liking scores provided to the sample added with the highest aroma amount (7.9 ± 0.3) compared to the sample with the lowest aroma amount and the base pudding formulation (6.7 ± 0.3; 5.9 ± 0.3, respectively). The main factor BMI was not significant (F = 0.69; *p* = 0.4). 

A significant sample effect (F = 5.7, *p* = 0.004) was found in the pudding modified with increasing amounts of butter aroma. The base formulation obtained the lowest liking score (5.8 ± 0.3) compared with A_Butter1 (6.7 ± 0.3) and A_Butter2 (7.4 ± 0.3), which did not significantly differ from each other. A significant BMI effect (F = 4.1, *p* = 0.04) on the liking score was also found, where a higher score was generally provided by overweight subjects (7.0 ± 0.2) compared with the control group (6.3 ± 0.3).

With regard to the pudding sample modified with the thickener agent, no significant sample and BMI effects were found on the liking scores. 

In Table 2, the results of the interaction of BMI × Sample in the three blocks of samples are reported. A significant interaction effect was found only in samples with a butter aroma. No significant differences in liking scores among samples by normal-weight subjects were found, whereas the butter aroma addition led to a significant increase in hedonic responses in obese subjects.

## 4. Discussion

The present study had the purpose of giving insight toward the investigation of cross-modal sensory interactions in normal-weight and overweight adolescents in order to increase desirable sensory characteristics in a vanilla pudding. The adolescents involved were also characterized for their gustatory function in order to assess differences in taste perception according to their body mass index. To the best of our knowledge, this is one of the first studies that has examined how multisensory perception, and the subsequent liking, occurs in adolescents with a different nutritional status.

The adolescents with a higher BMI were found to have a worse ability to correctly identify the taste qualities compared to the normal-weight subjects. This means that overweight adolescents need a higher stimulus concentration, such as a higher amount of sugar, in order to be able to detect and identify a specific taste quality. Consequently, they could have the need to perceive more intense sensory attributes in order to be satisfied from a sensory point of view. However, more intense attributes in terms of sweet, salty and fatty tastes are found in food rich in carbohydrates, fatty acid and sodium chloride, which usually characterize food overconsumed by overweight and obese subjects. Accordingly, these differences in the taste perception in adolescents with different BMIs have been previously reported [25,33]. However, it should be argued that literature data about taste perception according to the nutritional status in young subjects are poor and very controversial, with some authors not finding any differences in taste sensitivity between normal-weight and overweight children or adolescents [30,34], and other authors highlighting a higher responsiveness in subjects with a higher BMI [35]. These contradictory findings have also been depicted in adult subjects, especially with regard to the sweet perception, a taste quality that could be directly related to the phenomenon of weight gain [36]. Indeed, some research has indicated that obese subjects are less sensitive to sweetness [22,37,38], whereas other studies have reported no association between sweet taste acuity and weight status [39,40,41]. It is unclear whether, and if so how, the hedonic responses to sweet fat foods may be related to an altered taste threshold in obesity and how this altered perception might be related to hedonic responses. These inconsistent results could be due to several factors, such as the methodological approaches applied to investigate the gustatory function (i.e., chemical stimuli, preparations and concentrations), as well as the variability in subject classifications (i.e., age range, body weight).

The present data revealed several differences in the cross-modal sensory perception between the two groups of adolescents involved. With regard to the pudding samples modified with the vanilla aroma, the sensory attribute that was mainly affected by the aroma addition was the sweet taste. The ability of the aroma to signal sweet food in order to increase sweet taste perception has been widely reported using both water solutions [11,14,42] and model foods [13,43]. 

The overweight adolescents perceived the sweet taste in the base pudding formulation as significantly less sweet compared to the normal-weight subjects, confirming the results previously described about their weaker ability in correctly identifying this taste quality in the “taste strip” evaluation. Generally, the present data revealed that the vanilla aroma was able to increase the sweet taste perception, but only in a significant way for subjects with a higher BMI. This could be due to the higher “attention” and ability of overweight and obese subjects to perceive and respond to external food stimuli (e.g., all cues acquired by the five senses) compared with normal-weight subjects, such as olfactory stimuli, especially associated with pleasant food [27,44]. Indeed, overweight and obese subjects were previously found to have a better sensitivity to pleasant-food-related odors, which have a clear association to high-energy-dense food, such as chocolate [27]. Similarly, [26] showed that overweight children were more responsive to high energy food odorants, such as chocolate and beef odors, than normal-weight children. Concerning the hedonic data, the sample added with the higher aroma amount (Vanilla2) was the preferred sample in both groups of subjects, with no differences according to the BMI of the adolescents. It could be argued that increasing the perceived intensity of desirable sensory characteristics, such as a sweet taste, with additives such as flavor enhancers or aromas, could have a role in modulating the food reward towards food addiction [45]. However, the possibility of food addiction being a cause, co-morbidity or a consequence of obesity is still under discussion. Indeed, some researchers have suggested that the phenomenon of weight gain could result from an addictive tendency to consume food [46,47]. However, caution against merely attributing the development of this pathology to food addiction should be exercised [48]. It should also be considered that adding ingredients without any calories that are able to increase the sweet perception could decrease the amount of food eaten due to more satisfaction from a sensory point of view. This could be helpful in managing the amount of high-palatable food eaten, which is usually above the physiological need by adolescents [49].

Regarding the samples modified with the butter aroma, similar results were found. Indeed, the sweet taste perception was significantly increased by the aroma addition only in overweight subjects. The butter aroma could have been unconsciously associated with sweet food preparations and have led to an increase in this taste quality. In agreement with our results, it has been previously highlighted that the specific odors usually experienced with sucrose in sweet foods in western countries, such as strawberry, caramel and mint, are able to induce a sweetness enhancement [50]. Moreover, even if no real changes occurred in the texture dimension, samples with increasing concentrations of the butter aroma were perceived as being significantly creamier compared to the unmodified ones only in subjects with a higher BMI. Similar odor–texture interactions have been previously highlighted in food [16,51], with findings depicting that odors with fatty notes, such as coconut and butter, were able to increase the perceived thickness in low-fat stirred yoghurts [24]. These odor–texture interactions could have interesting practical implications in the development of food characterized by textures qualities that are able to increase satiety expectations, as well as actual satiating effects [16], without affecting the calorie amount. Beside the interesting odor–taste and odor–texture interactions, the hedonic evaluation was also significantly affected by the butter aroma addition. Indeed, in the overweight adolescents, the use of the aroma resulted in a significant increase in hedonic scores. Similar results were also previously highlighted in severe obese adults [28,29].

Samples modified by the thickener agent were perceived, as expected, as being significantly creamier by both groups of subjects. The addition of the xanthan gum also led to a decrease in the vanilla aroma perception. These results are in agreement with findings depicting a decrease in flavor perception due to modifications in the texture dimension. Indeed, some authors suggested that somatosensory stimuli can interact with taste and smell, modulating their perception [23,52,53]. In this context, pioneer data revealed that increasing the viscosity of a solution decreases, in some cases, both taste and flavor intensity [21,39]. These texture–odor and texture–taste interactions could be due to the limiting access of tastants and odorants to their receptors due to changes in the texture dimension.

The thicker agent addition did not significantly affect the hedonic responses, even if it should be argued that the scores slightly decrease with an increasing amount of the xanthan gum. This could be explained by the reduction in intensity of the perception of taste and aroma qualities characterizing the pudding samples.

## 5. Conclusions

The children involved in the present study showed both a different gustatory function and a different multisensory process involved in food perception in relation to their nutritional status. The overweight subjects showed a weaker ability in identifying the gustatory function, whereas they seemed to pay more attention to odor/stimuli signaling high-calorie products. Tricking our senses in the way of perceiving sensory attributes could be a promising strategy to develop innovative food formulations with, for example, a reduced sugar amount leading to a potential decrease in caloric intake, which could help to tackle the obesity epidemic.

## Figures and Tables

**Table 1:** Mean ± SEM taste strips scores for each taste quality and total taste score by BMI groups.

	Overweight (Mean ± SEM)	Normal-Weight (Mean ± SEM)	F
Sweet Score	2.96 ± 0.13	3.65 ± 0.15	11.66 ***
Bitter Score	2.64 ± 0.20	3.30 ± 0.27	4.73 *
Salty Score	2.60 ± 0.18	3.20 ± 0.20	5.18 *
Sour Score	2.08 ± 0.14	2.85 ± 0.15	13.89 ***
Total Taste Score (TTS)	10.28 ± 0.37	13.00 ± 0.42	23.40 ***

* and *** denote significant differences at 0.05 and 0.001, respectively.

**Table 2:** Mean liking scores (±SEM) by BMI in the three blocks of samples. Bold value shows significant differences in samples’ liking according to BMI.

Samples		Liking Scores (Mean ± SEM)		F
		Normal Weight	Overweight	
Modified with vanilla aroma				0.39 n.s
	Base_p	6.1 ^a^ ± 0.4	5.9 ^a^ ± 0.3	
	A_Vanilla1	6.4 ^a^ ± 0.5	6.9 ^b^ ± 0.4	
	A_Vanilla2	7.6 ^b^ ± 0.4	8.1 ^c^ ± 0.4	
Modified with butter aroma				4.5 **
	Base_ p	5.9 ± 0.4	5.8 ^a^ ± 0.3	
	A_Butter1	6.6 ± 0.5	6.7 ^b^ ± 0.3	
	A_Butter2	6.2 ± 0.4	8.5 ^c^ ± 0.4	
Modified with xanthan gum				
	Base_ p	6.1 ± 0.5	5.9 ± 0.4	0.08 n.s.
	T_Xanthan1	6.0 ± 0.5	5.7 ± 0.4	
	T_Xanthan2	5.6 ± 0.4	5.7 ± 0.4	

n.s. and ** denote not significant and significant differences at 0.01 among BMI groups, respectively. Different superscript letters (in column) depict significant hedonic differences among pudding samples in each BMI group.
